# Influence of Defect Number, Distribution Continuity and Orientation on Tensile Strengths of the CNT-Based Networks: A Molecular Dynamics Study

**DOI:** 10.1186/s11671-022-03656-w

**Published:** 2022-01-15

**Authors:** Xian Shi, Xiaoqiao He, Ligang Sun, Xuefeng Liu

**Affiliations:** 1 School of Civil Engineering, Suzhou University of Science and Technology, Suzhou, 215009 China; 2 Department of Architecture and Civil Engineering, City University of Hong Kong, Tat Chee Avenue, 999077 Kowloon Tong, Hong Kong; 3 Center for Advanced Structural Materials, City University of Hong Kong Shenzhen Research Institute, Shenzhen, 518057 China; 4 School of Science, Harbin Institute of Technology, Shenzhen, 518055 China; 5 Department of Mechanics and Aerospace Engineering, Southern University of Science and Technology, Shenzhen, 518055 China

**Keywords:** CNT-based network, Molecular dynamics simulation, Influence of defect, Mechanical performance

## Abstract

**Abstract:**

Networks based on carbon nanotube (CNT) have been widely utilized to fabricate flexible electronic devices, but defects inevitably exist in these structures. In this study, we investigate the influence of the CNT-unit defects on the mechanical properties of a honeycomb CNT-based network, super carbon nanotube (SCNT), through molecular dynamics simulations. Results show that tensile strengths of the defective SCNTs are affected by the defect number, distribution continuity and orientation. Single-defect brings 0 ~ 25% reduction of the tensile strength with the dependency on defect position and the reduction is over 50% when the defect number increases to three. The distribution continuity induces up to 20% differences of tensile strengths for SCNTs with the same defect number. A smaller arranging angle of defects to the tensile direction leads to a higher tensile strength. Defective SCNTs possess various modes of stress concentration with different concentration degrees under the combined effect of defect number, arranging direction and continuity, for which the underlying mechanism can be explained by the effective crack length of the fracture mechanics. Fundamentally, the force transmission mode of the SCNT controls the influence of defects and the cases that breaking more force transmission paths cause larger decreases of tensile strengths. Defects are non-negligible factors of the mechanical properties of CNT-based networks and understanding the influence of defects on CNT-based networks is valuable to achieve the proper design of CNT-based electronic devices with better performances.

**Graphical Abstract:**

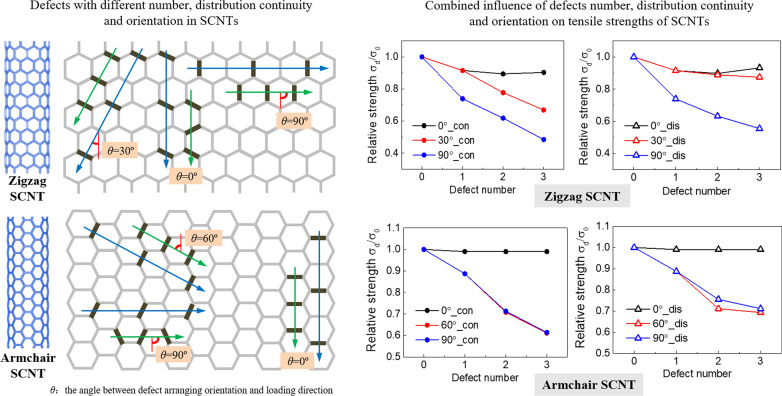

**Supplementary Information:**

The online version contains supplementary material available at 10.1186/s11671-022-03656-w.

## Introduction

Owing to the excellent mechanical and electronic properties [1–5], carbon nanotube (CNT) and graphene are promising candidates for the construction of the super-strong structure and carbon-based semiconductor devices. However, defects inevitably exist in CNT and graphene [6–8] and generally appear in the process of crystal growth and under the irradiation of particles and electrons [9, 10]. From the traditional view, defects in CNT and graphene may significantly deteriorate their properties. It is concluded that one- or two-atom vacancy defects reduce the failure stresses of CNTs by as much as 26% and markedly reduce the failure strains [11]. The thermal conductivity of CNTs also remarkably decreases with the increase in defect concentration [12]. Nevertheless, defects are reported to bring benefits in tailoring the properties of CNT/graphene and obtaining new functionality in recent years. Defects can be controlled to modify the transport properties and catalytic activity of the grapheme [13, 14], optimize the electrical performance of the graphene-based nano-devices [15], and obtain graphene clusters with electro-catalytic capability for better efficiency of the fuel cells [16]. The defect engineering of CNT or graphene has been proposed to achieve the material design and obtain different functionalities via the control of defects [9, 17].

Assembling CNTs into macro-structures, such as fibers, films and foams, is one of the important ways to achieve the real application of CNTs [18–20]. For instance, the CNT-based flexible electronic devices [21–24] are produced by fabricating CNTs into fibers or films through the solution deposition methods and chemical vapor deposition growth. Moreover, to achieve stronger connections, CNT-based networks and structures are fabricated by introducing covalent bonding between CNT units [25, 26]. Up to now, various CNT-based covalent networks have been proposed in theoretical investigations and achieved in experiments [27–32]. However, the performances of the CNT-based covalent networks are not as superior as the theoretical prediction. One of the vital reasons is that some connections of CNTs are not effective and defects exist in those CNT-based structures in practice.

Excellent deformability and mechanical properties are fundamental requirements for the practical applications of CNT-based networks, so identifying the influence of defects in CNT-based networks has crucial theoretical and practical meanings for CNT-based macro-structures and devices. At present, lattice defects at the junction area of the CNT connection have been investigated for the CNT-based networks in theoretical investigations [33, 34]. However, defects could also appear in the region of the straight CNT units after a series of physical and chemical treatments. But CNT units are mostly regarded as defect-free in the previous studies and the influence of the CNT-unit defect is not systematically investigated.

In this study, we investigate the influence of the CNT-unit defects on a CNT-based honeycomb network, namely super carbon nanotube (SCNT). Molecular dynamics (MD) simulations are carried out to systematically investigate the defect number, distribution continuity and orientation on the tensile strength of the CNT-based honeycomb networks.

## Methods

### Structures of the SCNT Models

**Fig. 1:**
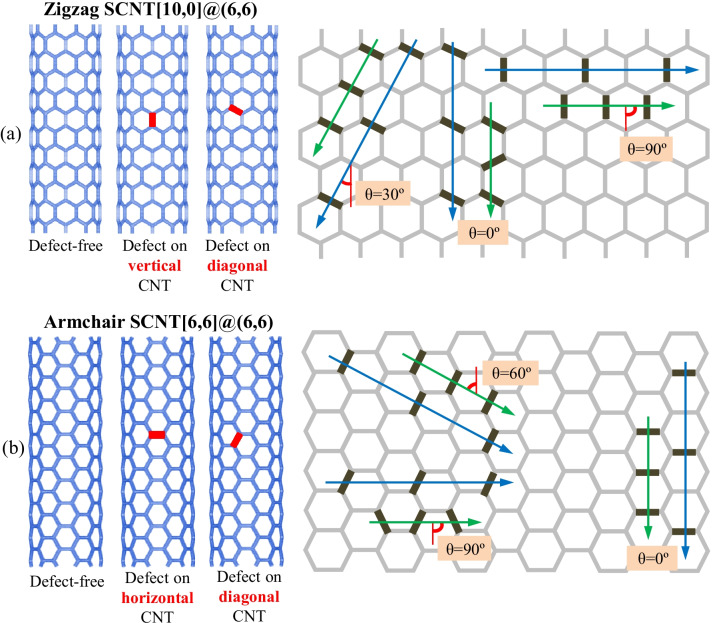
Distributions of defects in SCNTs with different numbers, orientations and continuity. **a** Zigzag SCNTs; **b** Armchair SCNTs

SCNT models are fabricated through the hierarchical assembly of CNTs and the detailed illustration can be seen from Additional file 1: Fig S1. According to the different assembly arrangements of CNTs, SCNT has two typical geometrical structures: zigzag structure and armchair structure, which are illustrated on the left side of Fig. 1a and b. The CNT unit with the chiral vector of (6,6) is adopted to fabricate armchair and zigzag SCNTs. Based on the hierarchical self-similar structure, SCNT is also represented by the chiral vector, with [10,0] for zigzag SCNT and [6,6] for armchair SCNT. The geometrical parameters for the SCNT models and CNT units are presented in Additional file 1: Table S1.

### Settings of Defects and Constructions of Defective SCNT Models

Due to the different orientations, CNT units in the armchair and zigzag SCNT structures are divided into two types, i.e., (1) the diagonal CNT and the vertical CNT for zigzag SCNT, and (2) the diagonal CNT and the horizontal CNT for armchair SCNT. Accordingly, the single-defect of the armchair and zigzag SCNTs corresponds to the location of the CNT units, which are diagonal and vertical single-defects for zigzag SCNTs and diagonal and horizontal single-defects for armchair SCNTs. As for the multi-defect cases, the defect number, distribution orientation and continuity are considered. In both zigzag and armchair SCNTs, there are three arranging orientations of the multi-defects, which are parallel/diagonal/vertical to the loading direction. As presented on the right side of Fig. 1a and b, the defect orientation is illustrated by the angle *θ*, which is the angle between the loading direction and the arranging direction of defects. Defects are distributed continuously (green arrows) or discontinuously (blue arrows) along with the specific directions. The defect number is up to three. To provide more quantitative information, the defect concentration of each model is determined corresponding to the defect number and presented in Additional file 1: Table S2. In the following parts, however, we will still use the defect number for discussion since it is simpler and more intuitive for readers to understand.

The defective SCNT models can be obtained by deleting a group of atoms and disconnecting several CNT units in the defect-free SCNT models (Additional file 1: Fig. S2). To clearly illustrate the different cases of the defect distribution, the name of the defective model is expressed as ‘*n*D/V/H_degree_con/dis.’ ‘$n$’ is the number of the defects and ‘D/V/H’ indicates the location of the defect (D is diagonal, V is vertical, H is horizontal). The ‘degree’ represents the angle *θ* and ‘con/dis’ shows the continuity of the defect distribution. For example, the SCNT model with two diagonal-CNT defects continuously distributed in parallel to the loading direction is named as ‘2D_0°_con’ and the model with the single-defect on the vertical CNT is named as ‘1V.’

### Molecular Dynamics Simulations

**Fig. 2:**
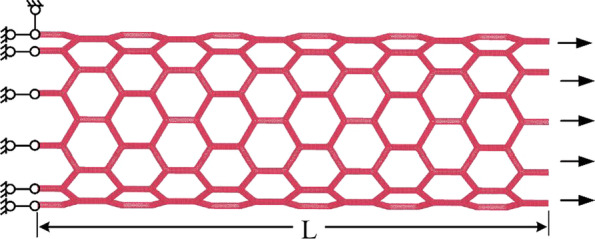
Tensile loading mode of the SCNT

Each resulting defective model experiences an energy minimization process to obtain the equilibrium configuration. After that, the axial tensile load is applied at one end of the SCNT model with the other end fixed (Fig. 2). The loading end moves at a constant rate of 0.1 Å/ps [28, 29, 35–37] and the radial direction of the SCNT model is free to shrink with the non-periodic boundary. By summarizing the counterforce at the fixed end of the SCNT, the axial tensile force of each model is obtained, which is based on Newton’s third law. Nosé–Hoover extended ensemble [38] is adopted and the temperature of each system is kept stable at 0.5 K during the entire simulation process. Adaptive Intermolecular Reactive Empirical Bond Order (AIREBO) potential [39] is applied to describe the interactions between carbon atoms, which is a popular and reliable force field for the simulations of carbon-based materials [40, 41]. All MD simulations in this study are carried out through the LAMMPS open-source code [42], a massively parallel simulator designed for large-scale atomic systems.

## Results and Discussions

Based on the results of MD simulations, the discussions about tensile strength and stress concentration of the defective SCNTs, as well as the underlying mechanisms of the influence of defect, are carried out and presented in the following three parts.

### Dependency of SCNT Strengths on Defect Number, Distribution Continuity and Orientation

**Fig. 3:**
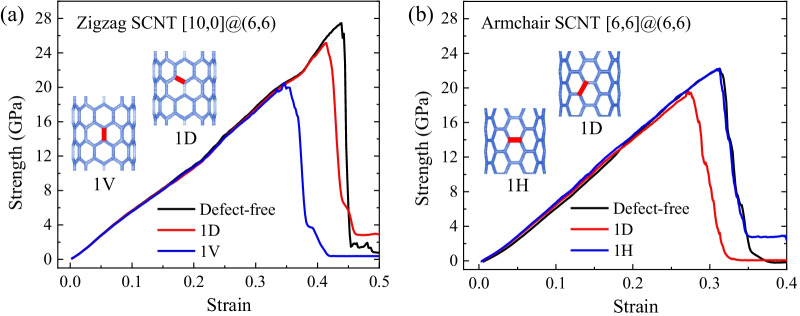
Stress–strain curves of the single-defect SCNTs compared with the defect-free SCNTs. **a** Zigzag SCNT; **b** Armchair SCNT

Tensile stress–strain curves of single-defect models are plotted in Fig. 3a and b, for armchair and zigzag SCNTs, respectively. Defect-free SCNT is set as a control group. It can be seen that most single-defects reduce the tensile strengths of SCNTs, while the defect on horizontal CNT brings almost no difference for the tensile curves of the armchair SCNT. Besides, the reductions of the strengths significantly rely on the defect location. For the zigzag SCNT, the single-defect on the diagonal CNT (model 1D) causes less strength reduction than the defect on the vertical CNT (model 1 V). In the armchair SCNT, the defect on the diagonal CNT (model 1D) is more influential than the one on the horizontal CNT (model 1H). Opposite to the great reduction of the ultimate strength, the shapes of the tensile curves are almost unchanged for all defective SCNTs, which means single-defects have a negligible effect on the tensile performances of the armchair and zigzag SCNT before fracture.

When the defect number increases, the strength reductions of defective zigzag SCNTs are generally more explicit than defective armchair SCNTs. The relative tensile strength is calculated for each model as the ratio of the strength of the defective model ${\sigma }_{d}$ to that of the defect-free model ${\sigma }_{0}$. Specific values of the tensile strength and relative tensile strength of both zigzag and armchair SCNTs are listed in Table 1. It can be seen that the largest drop of tensile strength is 51.5% for zigzag SCNTs, but the corresponding value is only 38.9% for armchair SCNTs.

**Table 1:** Tensile strengths and relative tensile strengths of defective zigzag and armchair SCNTs

Zigzag SCNTs			Armchair SCNTs		
Defective models	Strength (GPa)	Relative strength ${\sigma }_{d}/{\sigma }_{0}$	Defective models	Strength (GPa)	Relative strength ${\sigma }_{d}/{\sigma }_{0}$
Defect-free	27.45	1.000	Defect-free	22.22	1.000
1 V	20.28	0.739	1H	22.13	0.996
1D	25.12	0.915	1D	19.71	0.887
2D_0°_con	24.55	0.894	2H_0°_con	22.24	1.001
2D_0°_dis	24.64	0.898	2H_0°_dis	22.41	1.009
3D_0°_con	24.79	0.903	3H_0°_con	22.19	0.999
3D_0°_dis	25.58	0.932	3H_0°_dis	22.84	1.028
2D_30°_con	21.33	0.777	2D_60°_con	15.73	0.708
2D_30°_dis	24.41	0.889	2D_60°_dis	15.80	0.711
3D_30°_con	18.36	0.669	3D_60°_con	13.58	0.611
3D_30°_dis	23.98	0.874	3D_60°_dis	15.39	0.693
2V_90°_con	16.97	0.618	2D_90°_con	15.84	0.713
2V_90°_dis	15.35	0.632	2D_90°_dis	16.78	0.755
3V_90°_con	13.31	0.485	3D_90°_con	13.65	0.614
3V_90°_dis	15.24	0.555	3D_90°_dis	15.79	0.711

The distribution orientation and continuity of defects have a combined effect on the tensile strengths of defective SCNTs. In both zigzag and armchair SCNTs, the greatest drop of the tensile strength happens to the model with three continuously distributed defects. But the defects are vertically arranged in zigzag SCNT (3V_90° _con), while in armchair SCNTs it happens to both the vertical (3D_90°_con) and the diagonal direction (3D_60°_con). As for the smallest reduction of the tensile strength, model 3D_0°_dis of zigzag SCNT has only 6.8% reduction and all H-defect cases of armchair SCNTs bring almost no reduction for the tensile strength.

**Fig. 4:**
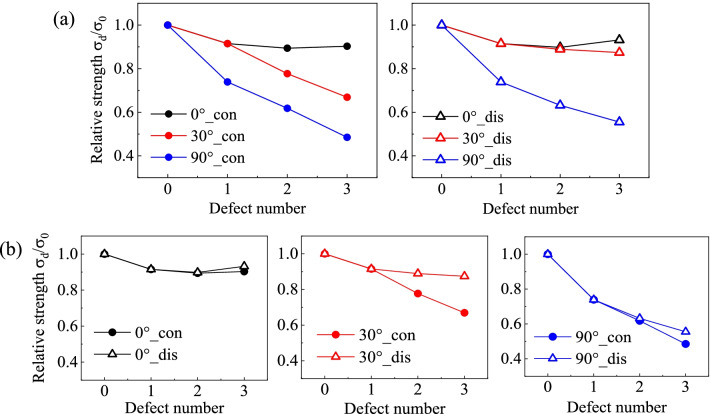
Variation of relative tensile strengths of defective zigzag SCNTs versus defect number. **a** Comparisons between different distribution orientations; **b** Comparisons between different distribution continuity

**Fig. 5:**
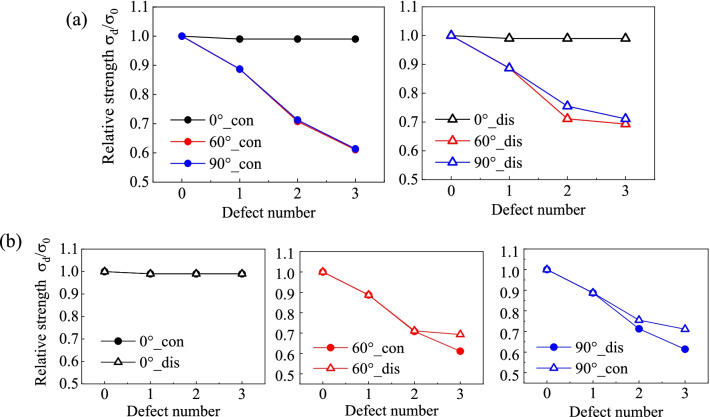
Variation of relative tensile strength of defective armchair SCNTs versus defect number. **a** Comparisons between different distribution orientations; **b** Comparisons between different distribution continuity

The relative tensile strength of each model is plotted versus the defect number in Figs. 4 and 5 for zigzag and armchair SCNTs, respectively. The defect number of 0 indicates the defect-free model. In Fig. 4a, tensile strengths of the defective zigzag SCNTs are compared between different defect orientations, for both continuous and discontinuous cases. It can be seen that the model with a larger angle to the loading direction has a larger reduction of tensile strength. As the increase in the defect number, the reductions induced by parallel distributed defects (0°) are all less than 10%, while the values for vertical orientations (90°) are almost triple for the same defect number. Moreover, the effect of orientation is more remarkable when defects are continuously distributed. The differences between parallel (0°) and diagonal distribution (30°) of defects are less than 5% when defects are discontinuously distributed, but the values turn to be 10 ~ 20% for continuous cases.

The differences induced by the defect continuity can be easily known from Fig. 4b. For each arranging orientation, the gap of each case is getting larger with the increase in defect number but the orientation-dependent characteristics are obvious. Significant strength differences can be observed for the diagonal distributed defects (30°), while the strength variations are insensitive to the distribution continuity of vertically (90°) and parallel (0°) distributed defects.

For armchair SCNTs, the combined influences of defect number, distribution continuity and orientation are distinct from the zigzag SCNT (Fig. 5). Diagonal and vertical distributions of defects possess approximating decreasing trends of tensile strengths as the defect number increases, while the parallel distributed defects bring no change of tensile strengths. For vertical and diagonal cases, the differences induced by the continuity turn to be obvious when the defects are more than one, but the vertically distributed defects have larger gaps than the diagonal cases.

### Different Stress Concentration Degrees and Modes Induced by Defects

**Fig. 6:**
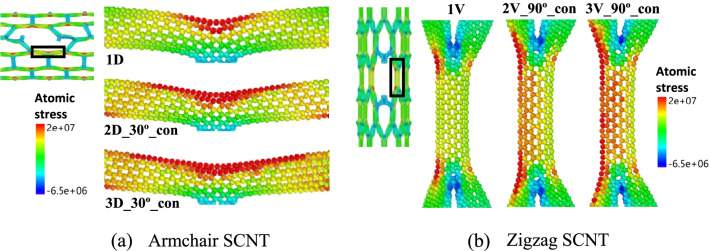
Local atomic stress distributions of the defective SCNTs. **a** Different defective armchair SCNTs under the same strain of 0.251; **b** Different defective zigzag SCNTs under the same strain of 0.285

The decreased strengths of defective SCNTs mainly result from the stress concentration around the defective positions. In Fig. 6, the local atomic stress distributions of the defective armchair and zigzag SCNTs are presented. It can be seen that under the same strain state the stress-concentration region expands dramatically for both armchair and zigzag SCNTs, meaning that more atoms are under high-stress states as the increase in defect number. To quantitatively evaluate the stress concentration degree induced by different defects, the stress level of the stress-concentrated area is identified for each model. The average stress of the stress-concentrated area is calculated and compared with that of the far-field area, then the ratio of these two average stresses is defined as the stress-concentration factor ${K}_{s}$. The detailed processes of the calculation are illustrated in Additional file 1: Fig. S3.

**Fig. 7:**
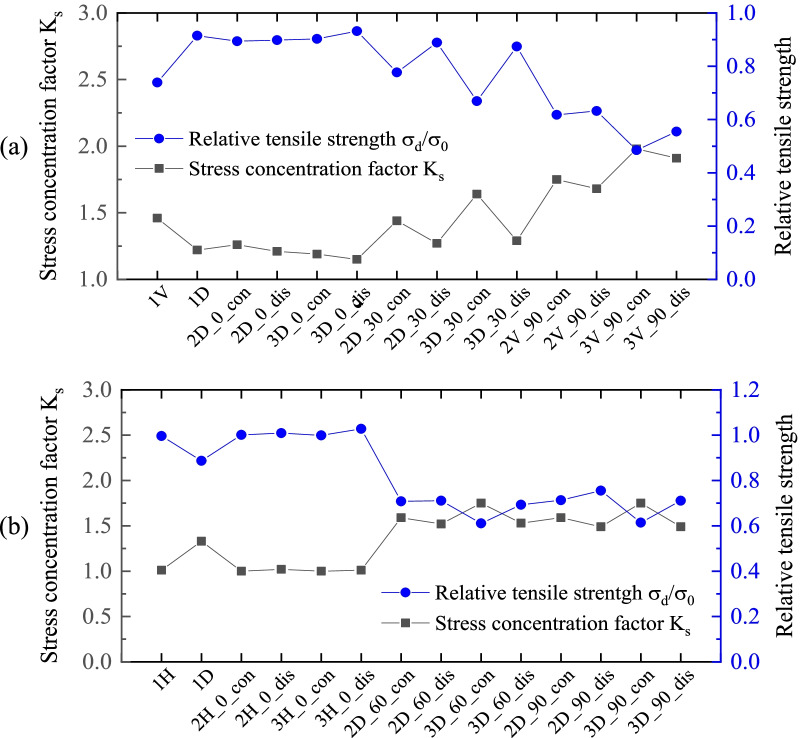
Stress concentration factors vs. relative tensile strengths for different defective SCNTs. **a** Zigzag SCNTs; **b** Armchair SCNTs

Specific values of ${K}_{s}$ are summarized in Table 2 for all models. To illustrate the relationship of stress and tensile strength in a more legible way, ${K}_{s}$ is plotted with the relative tensile strength in Fig. 7a and b, for zigzag and armchair SCNTs, respectively. It can be seen that the ${K}_{s}$ is inversely correlated with the trend of relative tensile strength for both armchair and zigzag SCNTs. Therefore, it can be concluded that, for defective SCNTs, higher values of stress concentration factor correspond to the smaller tensile strengths, which is consistent with the general rules of stress concentration.

**Table 2:** Stress concentration factors of defective models for zigzag and armchair SCNTs

Zigzag SCNT		Armchair SCNT	
Defective models	${K}_{s}$ via MD simulation	Defective models	${K}_{s}$ via MD simulation
Defect-free	1.00	Defect-free	1.00
1 V	1.46	1H	1.01
1D	1.22	1D	1.33
2D_0°_con	1.26	2H_0°_con	1.00
2D_0°_dis	1.21	2H_0°_dis	1.02
3D_0°_con	1.19	3H_0°_con	1.00
3D_0°_dis	1.15	3H_0°_dis	1.01
2D_30°_con	1.44	2D_60°_con	1.59
2D_30°_dis	1.27	2D_60°_dis	1.52
3D_30°_con	1.64	3D_60°_con	1.75
3D_30°_dis	1.29	3D_60°_dis	1.53
2V_90°_con	1.75	2D_90°_con	1.59
2V_90°_dis	1.68	2D_90°_dis	1.49
3V_90°_con	1.98	3D_90°_con	1.75
3V_90°_dis	1.91	3D_90°_dis	1.49

**Fig. 8:**
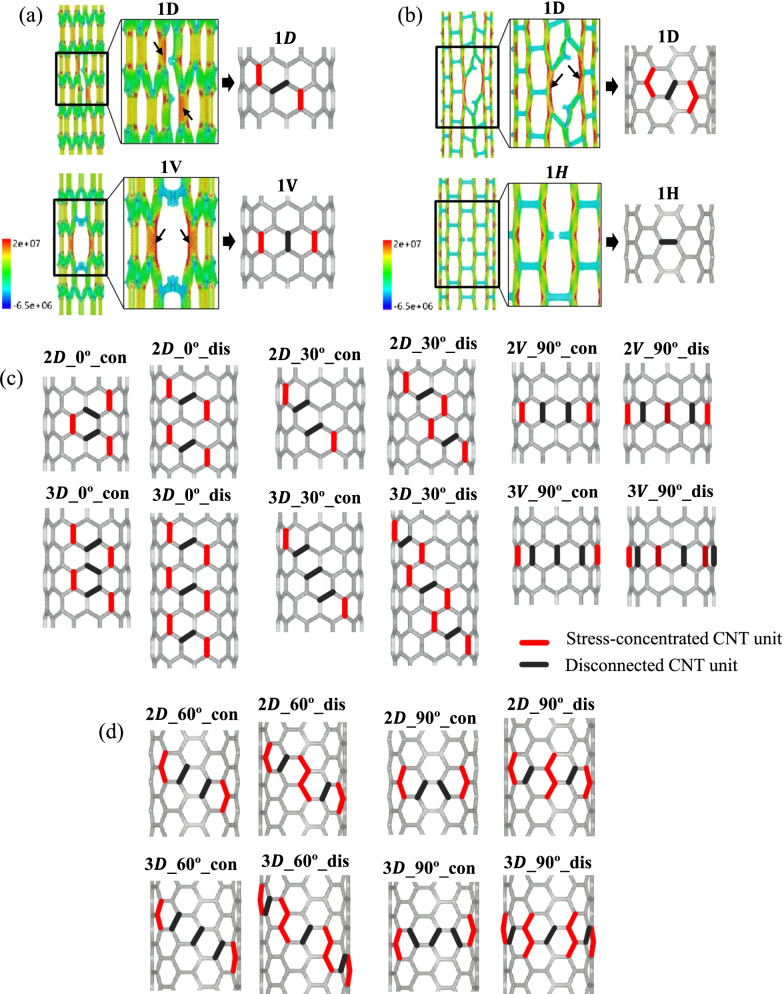
Stress concentration modes of the defective SCNTs. **a** Single-defect zigzag SCNTs; **b** Single-defect armchair SCNTs; **c** Multi-defect zigzag SCNTs; **d** Multi-defect armchair SCNTs

Moreover, the stress concentration level induced by defects is related to the stress concentration mode, which varies as the defect location, distribution continuity and orientation. According to the snapshots of MD simulations, the stress concentration modes of the defective armchair and zigzag SCNTs are illustrated in Fig. 8a and b. The disconnected CNTs are colored as black and the stress-concentrated CNTs are red-colored. It can be seen that the stress-concentration modes of both zigzag and armchair SCNTs change as the defect arrangements. For zigzag SCNTs, model 1 V has a symmetrical layout of the two stress-concentrated CNTs, while the stress-concentrated CNTs in model 1D are staggered. As for armchair SCNTs, explicit stress concentration can be observed for the 1D model, while there is no stress-concentrated area in the 1H model. That is why the disconnection of the horizontal CNTs does not affect the tensile strengths of armchair SCNTs.

The stress concentration modes of multi-defect SCNTs are also obtained based on the atomic stress distributions (Fig. 8c, d). As the increase in the defect number, stress concentration modes of the defective SCNTs are affected by both the distribution orientation and continuity of defects. For both armchair and zigzag SCNTs, the stress concentration regions extend with the increase in the defect number, and the extension generally follows the orientations of defect arrangement. However, the number of concentrated CNTs is not always proportional to the defect number but depends on the arranging continuity. For most discontinuous cases, the numbers of stress-concentrated CNTs proportionally increase as the defect number increases. Yet for continuously distributed cases, the number of concentrated CNTs keeps unchanging when the defect number increases. Besides, there are no stress-concentration phenomena for all horizontal defects of defective armchair SCNT models, no matter the settings of defect number and the distribution continuity. Thus, the stress-concentration modes of the armchair SCNTs with two or three horizontal defects are not presented in Fig. 8d.

The stress-concentration levels of the defective SCNTs are significantly related to their stress-concentration modes. The modes with more stress-concentrated CNTs lead to lower stress-concentration degrees because such SCNT models could bear the stress concentration more uniformly. Hence, when the defect number is fixed, the model with discontinuously distributed defects generally possesses lower stress-concentration degrees than one with continuously distributed defects, since the former one owns more stress-concentrated CNTs. However, some models with discontinuous defects have superpositions of the stress concentration at the same CNT unit, such as the zigzag SCNT models 2V_90°_dis and 3V_90°_dis in Fig. 8c. As a result, the stress concentration degrees of these models are much higher and the corresponding tensile strengths are much lower*.*

### Underlying Mechanisms of the Influence of Defect

#### Effective Crack Length

In the research of the macro-level honeycomb structure, the influence of defects has been systematically investigated and the defects are generally regarded as ellipse cracks [43, 44]. As a CNT-based honeycomb structure, the defects in this study can also be approximatively treated as ellipse cracks. According to the theory of fracture mechanics, it is known that the length of the ellipse crack primarily determines the stress-concentration level at the tip of the crack. Similarly, in this study, the influence of defects can be concluded from the comparisons of the crack lengths.

**Fig. 9:**
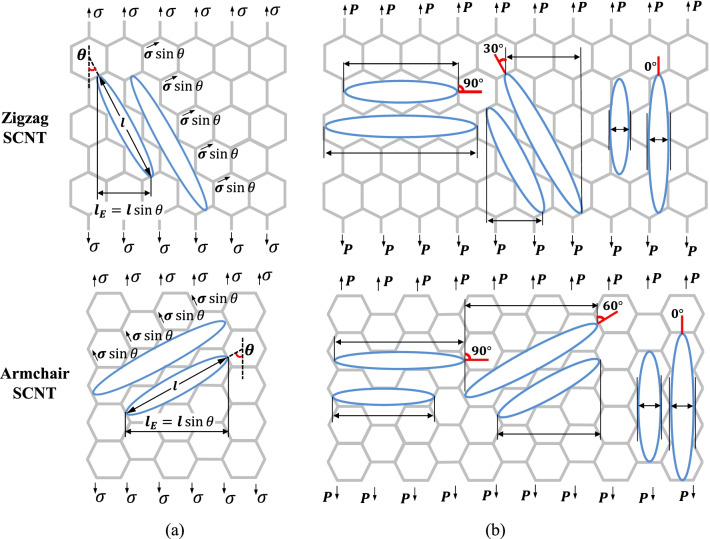
Illustration and comparison of the effective crack length of defective SCNTs. **a** Effective crack lengths of the defects in the armchair and zigzag SCNTs; **b** Effective length of the cracks formed by different oriented defects in the armchair and zigzag SCNTs

For qualitative comparisons, the crack length of each defect is roughly set as the longest distance of the damaged ranges, which is *l* in Fig. 9a. However, in the fracture mechanics, only the stress component that is perpendicular to the defect length effectively results in the stress concentration at the crack tip, which is the opening mode (Mode I) of the crack propagation. Therefore, the length that essentially determines the stress-concentration degree of the SCNT is the effective crack length, which is the projected length of the ellipse crack perpendicular to the loading direction (*l*_E_ in Fig. 9). In the study of the defective graphene [45], a similar approach of effective defect length is adopted for the discussion of stress concentration induced by defects.

By comparing the effective crack lengths of different defects (Fig. 9b), the reason for different stress-concentration degrees of defective SCNTs can be explained. In zigzag SCNT, the defect along with the vertical orientation (90°) always has the longest effective lengths when the crack length is fixed. Thus, the stress-concertation degrees of the models with vertically distributed defects are larger than those of the other cases, so the tensile strengths are much lower. For armchair SCNT, it should be noticed that the effective lengths of the vertical defects are approximate to that of the diagonal orientated defects for the same crack length. That is why these two cases often have approximate tensile strengths. Besides, when the defect orientation is parallel to the loading direction, the effective lengths of the cracks keep unchanged for both zigzag and armchair SCNTs, which is consistent with the invariable tendency of the stress-concentration degrees and tensile strengths of the most parallel distributed defects.

### Force Transmission Modes of SCNTs

**Fig. 10:**
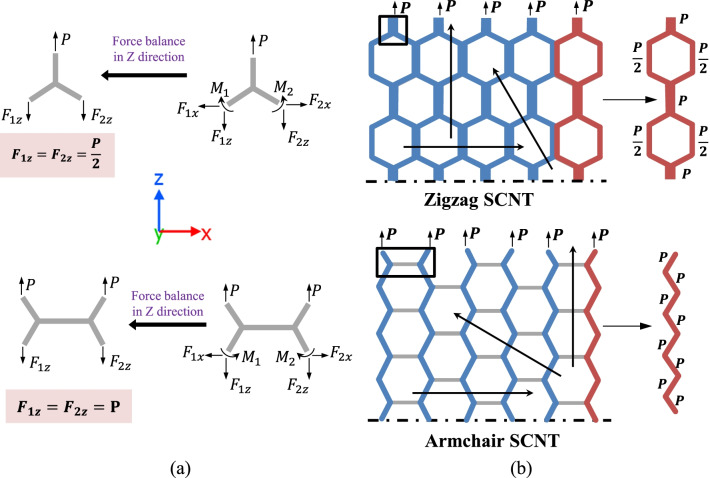
Demonstration of the force transmission modes of different SCNT structures. **a** Force balance analysis of free-body diagrams of zigzag and armchair SCNTs; **b** Force transmission modes of the zigzag and armchair SCNTs

The essential reason determining the influence of defects is the force transmission mode of the SCNTs. Based on the static force analysis, the force transmission modes of the armchair and zigzag SCNT are identified and illustrated in Fig. 10b. A detailed analysis of the free-body diagrams is presented in Fig. 10a. It can be seen that the mode of the zigzag SCNT is just the zigzag-like path that separates at the diagonal CNTs but merges at the longitudinal CNTs. Differently, the armchair SCNT has the CNT-bundle mode, in which the parallel chains comprised by diagonal CNTs are relatively independent for force transmission and the transverse CNT only plays a connection role.

Because of the different force transmission modes, the influence of defects is distinct for armchair and zigzag SCNTs. In zigzag SCNTs, the defect on vertical CNT cuts two force transmission paths simultaneously, whereas the defect on diagonal CNT only interdicts one path. Accordingly, more forces are released when the defects are located at the vertical CNTs, causing more decreases in the tensile strengths. In armchair SCNTs, only the defect on diagonal CNTs cut the real force transmission path, while the disconnection of transverse CNT has a neglectable effect on the force transmission network. That is why the defect on horizontal CNT brings no change for tensile strengths. Therefore, a defect at a more crucial position of the force transmission network causes a greater reduction of the tensile strength.

As for the multi-defect cases, the affected range of the force transmission path varies as the change of both defect number and orientation (illustrated as the black arrows in Fig. 10b). For zigzag SCNTs, the longitudinal distributed defects always induce the same width of the load-transfer path whatever the defect number is. Differently, the defects along with the diagonal and vertical directions proportionally widen the affected ranges of the force transmission network when the defect number increases. For armchair SCNTs, due to the ‘CNT-bundle’ force transmission mode, the defects along diagonal and vertical directions interdict the same width of the force transmission paths along loading direction, so the tensile strengths of these two directions are approximately the same in most cases. Defects along with the parallel direction do not cut any force transmission path, so they always do not affect the tensile strengths of armchair SCNTs.

## Conclusions

In this study, the influence of CNT-unit defects is investigated for the tensile strengths of the SCNTs via MD simulations. According to the above discussions, conclusions are obtained as follows.

1. The influence of defects is non-negligible for the performances of CNT-based networks and involves various factors including defect number, distribution continuity and orientation.
2. As the increase of the defect number, the tensile strengths of SCNTs generally keep decreasing and the decreasing rate significantly depends on the defect orientation and continuity.
3. Various stress-concentration modes are found for different arrangements of defects, which is notably correlated to the concentration degree and the tensile strength.
4. The influence of defects can be explained by the fracture mechanics. The cases with longer crack lengths along the loading direction lead to higher stress-concentration degrees and lower tensile strengths.
5. The force transmission mode of SCNTs is the essential mechanism determining the influence of the defect. Armchair and zigzag SCNTs have different force transmission modes, so the influence of defect is distinct for these two structures.
6. The existence of the defect breaks the force transmission network of SCNTs and larger affected ranges result in greater reductions of the tensile strength, which depends on defect arrangements.

The obtained conclusions provide a further view to the comprehensive understanding of the defect on CNT-based networks, which are valuable for the design of the CNT-based networks. With the proper optimization, CNT-based networks are believed to possess more favorable performances and better applications.

## Supplementary Information

**Additional file 1.** Supplementary materials.

## Data Availability

The datasets generated and/or analyzed during the current study are not publicly available since the data also forms part of an ongoing study, but are available from the corresponding author on reasonable request.
