# Supplementary material for: Influence of Defect Number, Distribution Continuity and Orientation on Tensile Strengths of the CNT-Based Networks: A Molecular Dynamics Study
Source: Nanoscale Res Lett. 2022 Jan 15;17:15. doi: 10.1186/s11671-022-03656-w (PMC8761213; doi:10.1186/s11671-022-03656-w)
Supplement: Supplementary file 1 — Additional file 1. Supplementary materials. [file 11671_2022_3656_MOESM1_ESM.docx]

**Supplementary Materials**

1. **Geometrical structure of the SCNT**

The assembly process of the SCNT model is presented in Fig. S1 (from the study of [1]). It can be seen that the structure of the SCNT is related to several geometrical parameters, including diameter and length for both CNT (d and *l* in Fig. S1) and SCNT (D and L in Fig. S1).

**

**

Fig. S1. Assembly process of the SCNT model [1]

Table S1 Lengths and diameters of the CNT and SCNT

| **SCNT models** | **CNT level** | | | **SCNT level** | | | |
| --- | --- | --- | --- | --- | --- | --- | --- |
|  | **Length (Å)** | **Diameter (Å)** | **Aspect ratio** | **Length (Å)** | **Diameter (Å)** | **Aspect ratio** |  |
| [6,6]@(6,6) | 33.06 | 8.14 | 4.06 | 189.42 | 597.31 | 3.15 |  |
| [10,0]@(6,6) | 32.56 | 8.14 | 4.01 | 180.01 | 526.46 | 2.92 |  |

The geometrical parameters of the two defect-free SCNT models in this study are presented in Table S1. These two models are fabricated by the CNT of (6,6) with the approximate length, so they have approximate sizes.

1. **Defective SCNT models**

In this study, the defects refer to the disconnections of the straight CNT units at certain areas of the SCNT networks. The defective SCNT model is obtained by deleting a group of atoms from the defect-free model, which can be seen from Fig. S2. The size of the deleted areas is chosen to avoid the mutual disturbance between the remaining two areas under loading.

Fig. S2. Obtaining defective SCNT models by deleting a group of atoms. (a) zigzag SCNT; (b) armchair SCNT. (A color version of this figure can be viewed online.)

In order to provide quantitative data, the defect concentration of each defective SCNT model is determined corresponding to the defect number. According to the definition of the defect concentration, the defect concentration of defective SCNT should be the ratio of the defective surface area to the whole surface area of the SCNT model, which is approximately equal to the ratio of the number of defective CNTs to the total CNT number in each SCNT. Because the SCNT models are comprised of the same CNTs with the same lengths. Hence, in this study, we calculate the defect concentration through the approximate way and the values are presented in Table S2.

Table S2 Defect concentration of SCNT models corresponds to the defect number

| SCNT models | CNT number | Defect concentration of defective SCNTs | | |
| --- | --- | --- | --- | --- |
|  |  | **n=1** | **n=2** | **n=3** |
| [6,6]@(6,6) | 360 | 2.7×10^-3^ | 5.5×10^-3^ | 8.3×10^-3^ |
| [10,0]@(6,6) | 330 | 3.03×10^-3^ | 6.06×10^-3^ | 9.09×10^-3^ |

1. **Calculation of the stress concentration factors** $K_{s}$**for defective SCNTs**

To quantificationally evaluate the stress concentration of different defective SCNTs, the stress concentration factor $K_{s}$ is calculated, which is the ratio of the average stress level of the stress-concentrated area to that of the far-field area. Corresponding locations of the stress-concentrated area and the far-field area are illustrated in Fig. S3 (a) and (b) for armchair and zigzag SCNT, respectively. The area used for the calculation of the average stress level can also be seen in Fig. S3 for armchair and zigzag SCNT (red-colored atoms). The chosen areas cover the major area of the stress concentration, which is settled by comparing with the concentrated area in Fig. 6 of the article body. For both the stress-concentrated area and the far-field area, the same numbers of atoms are taken for the calculation.

Fig. S3. Selected areas for the calculation of the stress concentration factor $K_{s}$. (a) armchair SCNT；(b) zigzag SCNT. (A color version of this figure can be viewed online.)

***Reference***

*[1] X. Shi, X. He, L. Wang, L. Sun, Hierarchical-structure induced adjustable deformation of super carbon nanotubes with radial shrinkage up to 66%, Carbon. 125 (2017) 289–298.*
